# RNA-binding proteins potentially regulate the alternative splicing of cell cycle-associated genes in proliferative diabetic retinopathy

**DOI:** 10.1038/s41598-024-57516-x

**Published:** 2024-03-20

**Authors:** Ning Yang, Ningzhi Zhang, Guojing Lu, Siyu Zeng, Yiqiao Xing, Lei Du

**Affiliations:** Department of Ophthalmology, Renmin Hospital of Wuhan University, Wuhan, China

**Keywords:** Diabetes complications, Gene regulation

## Abstract

RNA-binding proteins (RBPs) contribute to the pathogenesis of proliferative diabetic retinopathy (PDR) by regulating gene expression through alternative splicing events (ASEs). However, the RBPs differentially expressed in PDR and the underlying mechanisms remain unclear. Thus, this study aimed to identify the differentially expressed genes in the neovascular membranes (NVM) and retinas of patients with PDR. The public transcriptome dataset GSE102485 was downloaded from the Gene Expression Omnibus database, and samples of PDR and normal retinas were analyzed. A mouse model of oxygen-induced retinopathy was used to confirm the results. The top 20 RBPs were screened for co-expression with alternative splicing genes (ASGs). A total of 403 RBPs were abnormally expressed in the NVM and retina samples. Functional analysis demonstrated that the ASGs were enriched in cell cycle pathways. Cell cycle-associated ASEs and an RBP–AS regulatory network, including 15 RBPs and their regulated ASGs, were extracted. Splicing factor proline/glutamine rich (SFPQ), microtubule-associated protein 1 B (MAP1B), heat-shock protein 90-alpha (HSP90AA1), microtubule-actin crosslinking factor 1 (MACF1), and CyclinH (CCNH) expression remarkably differed in the mouse model. This study provides novel insights into the RBP–AS interaction network in PDR and for developing screening and treatment options to prevent diabetic retinopathy-related blindness.

## Introduction

Diabetic retinopathy (DR), a complication that affects up to 30% of patients with diabetes, involves retinal neurodegeneration and is a leading cause of vision loss in middle-aged and elderly people^1^. Without treatment, non-proliferative DR advances to proliferative DR (PDR), which involves retinal neovascularization (RNV).

Complex interrelated pathophysiological mechanisms triggered by hyperglycemia underlie PDR development. Previous studies reported that genetic and epigenetic factors, increased free radical production, advanced glycosylation end-products, inflammatory factors, and vascular endothelial growth factor (VEGF) contribute to PDR development^2^. The pathophysiological mechanisms underlying DR must be elucidated to develop novel screening and treatment options to prevent DR-related blindness.

RNA-binding proteins (RBPs) bind RNAs through at least one globular RNA-binding domain and affect the fate or functions of the bound RNAs^3^. They regulate transcription-dependent and -independent gene expression and molecular functions, such as transcription, RNA processing, mRNA export, and RNA decay^3^. Therefore, abnormal expression or dysfunction of RBPs may contribute to the genesis and progression of various diseases^3,4^.

Many RBPs play important roles in eye development and disease pathogenesis, suggesting their use as targets to treat diseases. For instance, RNA-binding motif protein 24 can directly bind to the 3ʹ-untranslated region (3ʹ-UTR) of Sox2 mRNA, a key transcription factor, thereby mediating eye development^5^. HuR, an RBP that can stabilize the mRNA expression of several genes, such as VEGF and tumor necrosis factor-α, can also be used as a target to treat DR, suggesting the potential use of RBPs as novel markers^6^. Moreover, the RBP galectin-1 is reportedly involved in angiogenesis and RNV^7,8^. These findings suggest the need to further explore the functions of RBPs differentially expressed (DE) in PDR and elucidate the underlying mechanisms to develop and optimize treatments for this disease.

RBPs in retinal tissue exert multiple functions, such as acting as trans-acting splicing factors and enhancing or repressing splicing events^9^. Pre-mRNA alternative splicing (AS) is an important source of protein diversity in organisms. Abnormal post-transcriptional RNA splicing in retinal tissues may contribute to the development of retina-related diseases^10,11^. Specifically, abnormal AS of VEGF in retinal tissues promotes DR progression by altering the relative ratio of VEGF antiangiogenic and proangiogenic protein isomers^12,13^. Such changes can mediate disease progression^14^. Thus, abnormal AS could serve as a potential target to treat PDR. Metformin inhibits DR development by modulating the AS of VEGFA^15^. AS events (ASEs) regulate gene expression, but their roles in PDR remain unclear to date.

CUGBP Elav-like family member 1 mediates fiber cell morphology by regulating the mRNA expression of the F-actin crosslinking factor Actn2 and the AS of the membrane-organization factor beta-spectrin, suggesting that post-transcriptional regulatory RBPs have evolved conserved functions to influence vertebrate oculogenesis^16^. Splicing factor proline/glutamine rich (SFPQ) binds to the mRNA of microphthalmia-associated transcription factor (MITF) and regulates retinal pigment epithelium cell proliferation by modulating the AS of MITF^17^. The RBP embryonic lethal abnormal vision like 1 regulates the AS of the eukaryotic translation initiation factor 4E nuclear import factor 1 to promote postnatal angiogenesis^18^. However, a systemic analysis of the correlation between RBPs and DE ASEs during PDR development remains lacking to date.

We hypothesized that DE RBPs in the retina affect PDR development by modulating the AS of downstream genes. The aim of this study was to identify DE genes (DEGs) in the neovascular membranes (NVMs) and retinas of patients with PDR.

## Materials and methods

### Retrieval and processing of public data

We downloaded the public transcriptome dataset GSE102485 from the Gene Expression Omnibus (GEO) database. The Sequence Read Archive (SRA) was converted to fastq format using the NCBI SRA Tool fastq-dump. From the dataset GSE102485, we downloaded 17 neovascular membrane (NVM) tissue samples from patients with type II diabetes (T2D) and PDR, 3 NVM tissue samples from patients with type I diabetes (T1D) and PDR, and 3 normal retinas from donated eyes of normal individuals (Nor-R)^19^. Data about human tissue samples used in the study all come from publically available datasets. Raw reads were trimmed using the FASTX-Toolkit (v.0.0.13; http://hannonlab.cshl.edu/fastx_toolkit/). Clean reads were evaluated using FastQC (http://www.bioinformatics.babraham.ac.uk/projects/fastqc). The specific sampling sites of 17 neovascular membrane (NVM) tissue included 4 hemal arch, 3 nasal side, 4 whole part, 2 hemal arch part and optic disc, 2 optic disc, and 2 retinas. After combining data according to tissue sources, we obtained 6 NVM samples by combining the data from GSE102485 based on the data size of 20 NVM samples (17 T2D and 3 T1D) (Supplementary Table 1). In the retinas of patients with PDR (PDR-R), one sample was obtained by combining the original data of retinas from two patients with T2D. Because of an outlier sample (SRR5925098) during sample correlation analysis and sample cluster analysis, two samples of retinas from normal people were used in the Nor-R group (Supplementary Fig. 1).

### Read alignment and DEG analysis

HISAT was used to align clean reads to the mouse genome, allowing for four errors^20,21^. Uniquely mapped reads were ultimately used to calculate the read number and fragments per kilobase of exon model per million mapped reads (FPKM) for each gene. The FPKM method was used to assess gene expression. DEseq2 was used to analyze gene differential expression. This software models the original reads and uses a scale factor to explain the difference in library depth. Then, it estimates the gene dispersion and reduces these estimates to generate accurate dispersion estimates to model the read count. This software was used to fit the negative binomial distribution model, and the hypothesis was verified using the Wald test or the likelihood ratio test. Genes showing a fold-change (FC) ≥ 2 or ≤ 0.5) and false discovery rate (FDR) ≤ 0.01 were considered DE.

### AS analysis

The ASEs and regulated ASEs (RASEs) among the samples were defined and quantified using the ABLas pipeline as described previously^22,23^. Briefly, the ABLas detection of 10 types of ASEs was based on splice junction reads, including exon skipping (ES), alternative 5ʹ splice site (A5SS), alternative 3ʹ splice site (A3SS), intron retention (IR), mutually exclusive exons (MXE), mutually exclusive 5ʹ-UTRs (5pMXE), mutually exclusive 3ʹ-UTRs (3pMXE), cassette exon, A3SS&ES, and A5SS&ES. The significance of pair comparisons was determined using Fisher’s exact test, with the alternative and model reads of the samples as input data. The RASE ratio was obtained by calculating the changed ratio of alternatively spliced to constitutively spliced reads among the samples. RASE ≥ 0.2 and *P*-value ≤ 0.05 were set as the thresholds for RASE detection. The significance of the ratio alteration of ASEs was evaluated using Student’s t-test. Events that were significant at a *P*-value cutoff of 0.05 were considered non-intron retention (NIR) RASEs.

### Functional enrichment analysis

Gene Ontology (GO) terms and Kyoto Encyclopedia of Genes and Genomes (KEGG) pathways were identified using KOBAS 2.0 to determine the functional categories of the DEGs^24^. The hypergeometric test and Benjamini–Hochberg FDR control procedure were used to define the enrichment of each term. Functional enrichment analysis of the sets of selected genes was conducted using Reactome (http://reactome.org) pathway profiling.

### Animal validation experiment

The oxygen-induced retinopathy (OIR) mouse model, which is the most widely used animal model to surrogate ischemic retinopathies, such as PDR, was used in the validation experiment^25^. OIR was established in accordance with the protocol described by Smith et al.^8,26^. Briefly, postnatal day 7 pups, together with their nursing mothers, were continuously exposed to 75% oxygen in a hyperoxic tank for 5 days. On postnatal day 12, the mice were returned to ambient air for 5 days and then sacrificed to harvest their retinas on postnatal day 17. Normal control pup mice were exposed to ambient air before being sacrificed on postnatal day 17. Abnormal RNV, observed by whole-mount retinal immunostaining, was considered a marker of successful OIR model construction (Supplementary Fig. 2). Retinas from mouse pups were used to validate the expression difference between normal and OIR mice (n = 3). Two retinas from the same mouse were used as a group for later experiments.

All C57BL/6J mice used were obtained from the Laboratory Animal Center of Wuhan University, and all related experiments were approved by the Committee on the Ethics of Animal Experiments of Wuhan University. All procedures were performed in strict accordance with the ARRIVE guidelines and national animal experiment guidelines and conformed to the Association for Research in Vision and Ophthalmology Statement for the Use of Animals in Ophthalmic and Vision Research.

### Quantitative RT-PCR

The validity of the DE lncRNAs was evaluated using qRT-PCR. The RNA integrity of each sample was evaluated and quantified using 1.5% agarose gel electrophoresis and spectrometry. Purified RNA was reverse-transcribed to generate DNA using a PrimeScript RT Reagent Kit (Takara, Shiga, Japan). qRT-PCR was performed using the TB Green Fast qPCR Mix (Takara) and specific primers (Table 1). The amplification conditions were as follows: pre-denaturation at 95 °C for 5 min, followed by 40 cycles of denaturation at 95 ℃ for 15 s and annealing and extension at 60 ℃ for 30 s. The 2^−ΔΔCT^ method was used to determine relative gene expression, which was normalized against the level of GAPDH.

**Table 1:** Primers.

Gene	Sequence (5ʹ–3ʹ)
Macf1-F	GCTGGTGAACTCCGTTAA
Macf1-R	TTGGTGGCACTGTGAATT
Sfpq-F	CCTTCCTGTGTTACTTCTATG
Sfpq-R	CTATGTAGCCTAAGCAACTATC
Hsp90aa1-F	ACGCTCTATCAACCTCTTG
Hsp90aa1-R	TGCTTACCTTCATTCCTTCT
Map1b-F	CAGTGTCATCATAGCCATTG
Map1b-R	AGACCATTGAGAAGACCATC
Tpt1-F	CCATACAACACCAGGACTTA
Tpt1-R	ATGCTCAACCAGGCTCTA
Lgals1-F	TGTGCTGAACCTGGGAAA
Lgals1-R	CCATCTTCCTTGGTGTTACA
mus-GAPDH-F	GGAGATGCTCAGTGTTGG
mus-GAPDH-R	TGACAATGAATACGGCTACA
CCAR1-M-F	GCAAATGAAAATCAGTTATTT
CCAR1-AS-F	CCAAAGGCATTCAGTTATTT
CCAR1-M/AS-R	AGCCTCCAGAACTGTGAGAAGTAA
CCPG1-M-F	TAGTGGAGGACTTGTCTGAC
CCPG1-AS-F	TCACAATGTGCTTGTCTGAC
CCPG1-M/AS-R	ACACTACCAGAGTATAAGAG
CCNH-M/AS-F	ACTATGCTATAGAATTGTGA
CCNH-AS-R	AATATTTTTGCCCTGACCTT
CCNH-M-R	TTAGTAGAGACCCCTGACCTT

F: forward; R: reverse.

### Western blot analysis

Retinas were homogenized with a lysis buffer which contained 50 mM Tris–HCl, 150 mM NaCl, 1% NP-40, 0.1% SDS, 0.5% sodium deoxycholate, 1 mM EDTA, 1 mM PMSF, and a protease inhibitor cocktail. A bicinchoninic acid (BCA) protein assay kit (EpiZyme, Shanghai, China) was used to measure the concentration of total protein in accordance with the manufacturer's instructions. Protein was separated in 10% SDS-PAGE and transferred to polyvinylidene fluoride (PVDF) membranes. Membranes were incubated with TBST buffer (20 mM Tris-buffered saline and 0.1% Tween-20) containing 5% non-fat milk powder for 1 h at room temperature. Membranes were cut based on the protein molecular weight of the target molecules to prevent nonspecific stray band interference and then incubated with primary antibodies against SFPQ (1:10,000, Abcam, Cambridge, England) and GAPDH (1:5,000, HUABIO, Hangzhou, China), followed by incubation with HRP-conjugated secondary antibody (anti-rabbit 1:3,000, Servicebio, Wuhan, China) for 2 h at room temperature. Bound secondary antibodies were detected using an enhanced chemiluminescence reagent (Bio-Rad, CA, USA) and analyzed using Image-J software (National Institutes of Health, USA).

### Statistical analysis

Principal component analysis (PCA) was performed using the factoextra package in R to display the clustering of samples with the first two components. An in-house script (sogen) was used to visualize the next-generation sequence data and genomic annotations after normalizing the reads by the tags per million for each gene in the samples. Clustering analysis based on Euclidean distance was performed using the pheatmap package in R. Two groups were compared using Student’s *t*-test. The experimental data were presented as means ± SEM, and a *P*-value of < 0.05 was considered statistically significant.

## Results

### Gene expression profile of NVM and retinas from patients with PDR

**Figure 1:**
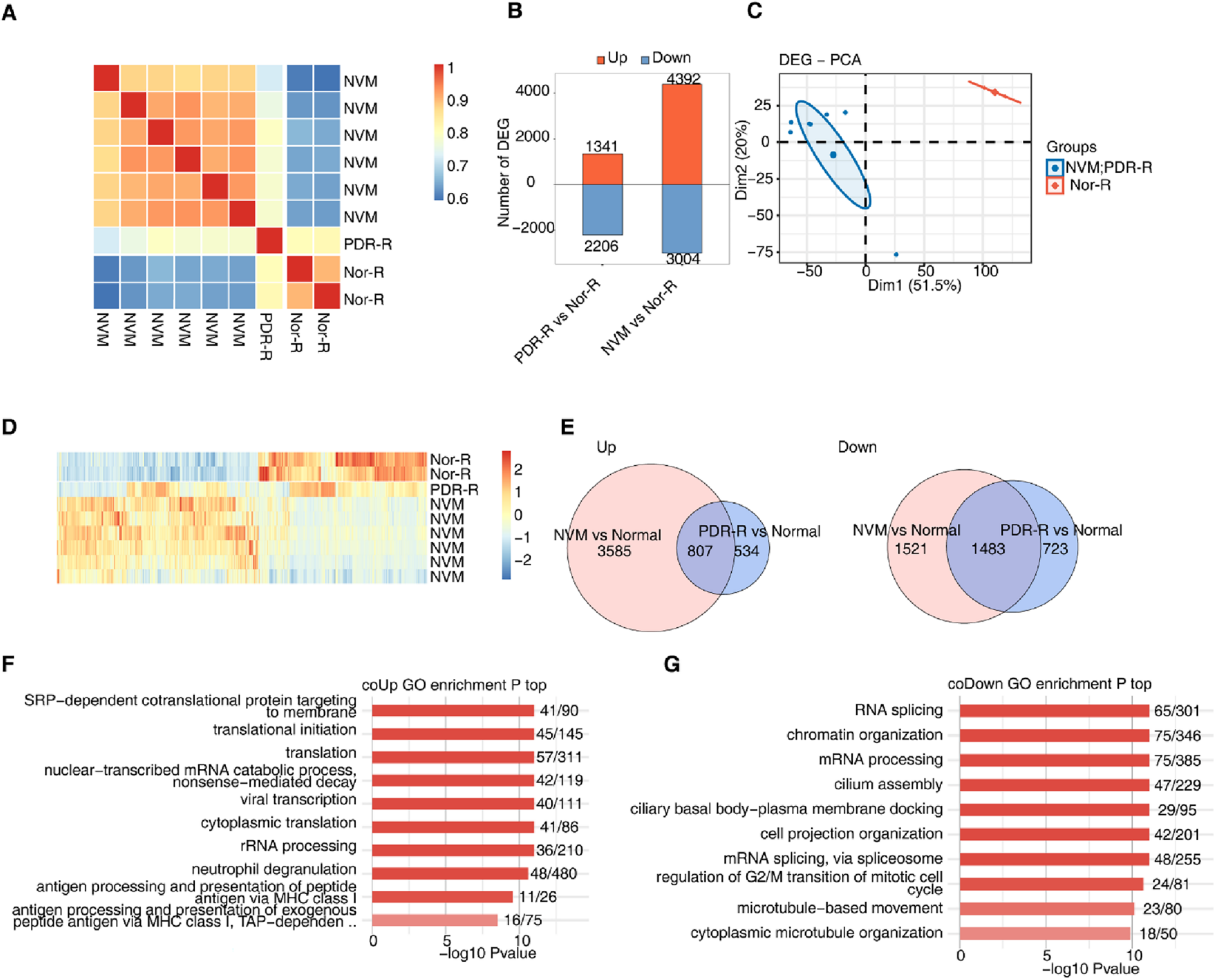
Gene expression profile of NVMs and retinas from patients with proliferative diabetic retinopathy. (**A**) Hierarchical clustering heatmap showing correlation between samples from the NVM, PDR-R, and normal groups based on the FPKM values of all expression genes. (**B**) Bar plot showing all DEGs between samples from the NVM, PDR-R, and normal groups with DESeq2. False discovery rate ≤ 0.05 and fold-change ≥ 2 or ≤ 0.5. (**C**) PCA based on FPKM values of all DEGs. The ellipse for each group is the confidence ellipse. (**D**) Hierarchical clustering heatmap displaying the expression levels of all DEGs. (**E**) Venn diagram of the gene ID showing the co-upregulated DEGs (left) and co-downregulated DEGs (right) between samples from the NVM and PDR-R groups. (**F**) Bar plot showing the most enriched GO biological processes of the co-upregulated genes. (**G**) Bar plot showing the most enriched GO biological processes of the co-downregulated genes. DEGs, differentially expressed genes; FPKM, fragments per kilobase of transcript per million fragments mapped; GO, Gene Ontology; NVM, neovascular membranes; PCA, principal component analysis; PDR-R, retinas from patients with PDR.

Initially, we analyzed the gene expression profiles in the NVM, PDR-R, and Nor-R groups. The hierarchical clustering heatmap showed a correlation among the genes from the NVM, PDR-R, and Nor-R groups based on their FPKM values (Fig. 1A). A total of 3547 DEGs (1341 upregulated and 2206 downregulated) were identified between the PDR-R and Nor-R groups (Fig. 1B). Moreover, 7396 DEGs (4392 upregulated and 3304 downregulated) were found between the NVM and Nor-R groups (Fig. 1B). PCA results showed similar DEGs between the NVM and PDR-R groups (Fig. 1C,D). Specifically, 807 genes were co-upregulated and 1483 genes were co-downregulated in the PDR-R and NVM groups (Fig. 1E). GO functional analysis indicated that the co-upregulated DEGs were enriched in many GO pathways, the top 10 of which were as follows: SRP-dependent co-translational protein targeting to membrane; translational initiation; translation; nuclear-transcribed mRNA catabolic process; nonsense-mediated decay; viral transcription; cytoplasmic translation, rRNA processing; neutrophil degranulation; antigen processing and presentation of peptide antigen via major histocompatibility complex (MHC) class I; and antigen processing and presentation of exogenous peptide antigen via MHC class I, TAP-dependent (Fig. 1F). GO functional analysis revealed that the co-downregulated DEGs were enriched in several GO pathways, the top 10 of which were as follows: RNA splicing; chromatin organization; mRNA processing; cilium assembly; ciliary basal body–plasma membrane docking; cell projection of organization; mRNA splicing via spliceosome; regulation of G2/M transition of mitotic cell cycle; microtube-based movement; and cytoplasmic microtubule organization (Fig. 1G). KEGG pathways of the co-upregulated and co-downregulated genes are shown in Supplementary Fig. 3A,B.

### Abnormal AS patterns of genes in NVM and retinas from patients with PDR

**Figure 2:**
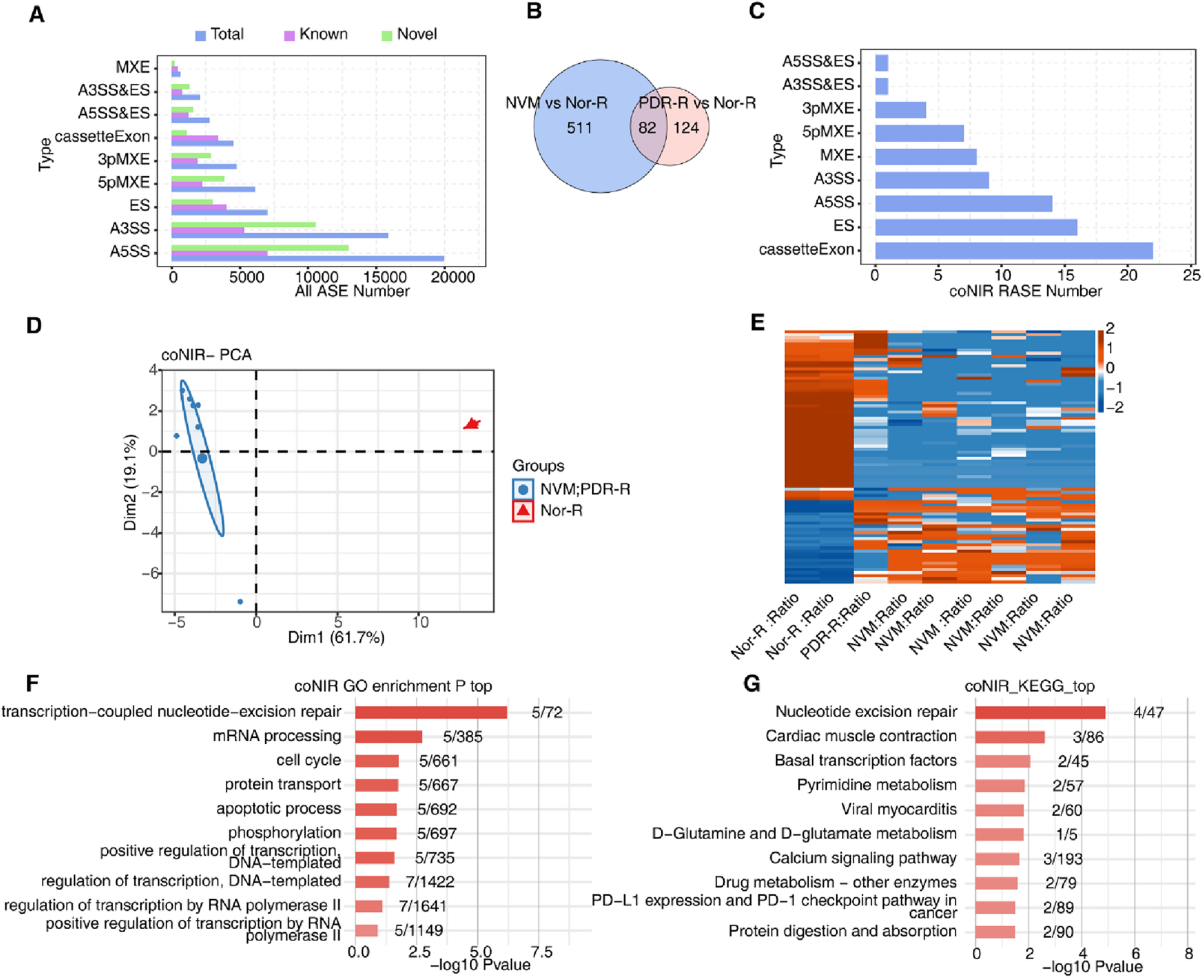
Abnormal alternative splicing patterns of genes in NVMs and retinas from patients with PDR. (**A**) Bar plot showing the number of all significantly regulated ASEs between samples from the NVM, PDR-R, and normal groups. X-axis: ASE numbers. Y-axis: Different types of ASEs. (**B**) NIR RAS (no-intron) filtering should have detectable splice junctions in all samples, and at least 80% of samples should have ≥ 10 splice junction supporting reads. Venn diagram of the gene ID showing the overlapped RAS (coNIR RAS) between samples from the NVM and PDR-R groups. (**C**) Bar plot showing the number of all significantly regulated ASEs (coNIR RAS) between NVM and PDR-R and normal samples. X-axis: coNIR RASE number. Y-axis: Different types of ASEs. (**D**) PCA was based on the splicing ratio of all coNIR RAS. The ellipse for each group was a confidence ellipse. (**E**) Hierarchical clustering heatmap of all significant coNIR RAS based on splicing ratio. (**F**) Bar plot showing the most enriched GO biological processes of all significant coNIR RAS. (**G**) Bar plot showing the most enriched KEGG pathway results for all significant coNIR RAS. ASEs, alternative splicing events; coNIR, co-expressed non-intron retention; GO, Gene Ontology; KEGG, Kyoto Encyclopedia of Genes and Genomes; NVM, neovascular membranes; PCA, principal component analysis; PDR, proliferative diabetic retinopathy; PDR-R, retinas from patients with PDR; RAS, regulated alternative splicing; RASE, regulated alternative splicing events.

After AS analysis, we identified nine types of differential ASEs: MXE, A3SS&ES, A5SS&ES, cassetteExon, 3pMXE, 5pMXE, ES, A3SS, and A5SS. These ASEs contained many known and novel ASEs (Fig. 2A). We found 593 DE ASEs between NVM and Nor-R and 206 DE ASEs between PDR-R and Nor-R (Fig. 2B). Moreover, 82 DE ASEs were identified in the PDR-R and NVM groups compared with the Nor-R group (Fig. 2C). Based on the percent-splice-in (PSI) value of the AS timepoint in every sample, PCA and hierarchical clustering heatmap showed that the six NVM samples and one PDR-R sample were enriched together, whereas NOR-R was enriched separately (Fig. 2D,E). GO and KEGG analysis results showed that these DE ASEs were enriched in the following pathways: transcription-coupled nucleotide-excision repair; pyrimidine metabolism; mRNA processing; cell cycle; protein transport; apoptotic process; phosphorylation; positive regulation of transcription, DNA-templated; regulation of transcription, DNA-templated; regulation of transcription by RNA polymerase II; basal transcription factors; positive regulation of transcription by RNA polymerase II, and so on (Fig. 2F,G). Additional data regarding the abnormal AS patterns of genes in NVM and PDR-R are shown in Supplementary Fig. 4.

### Co-expression analysis between RBPs and ASGs in NVM and retinas from patients with PDR

**Figure 3:**
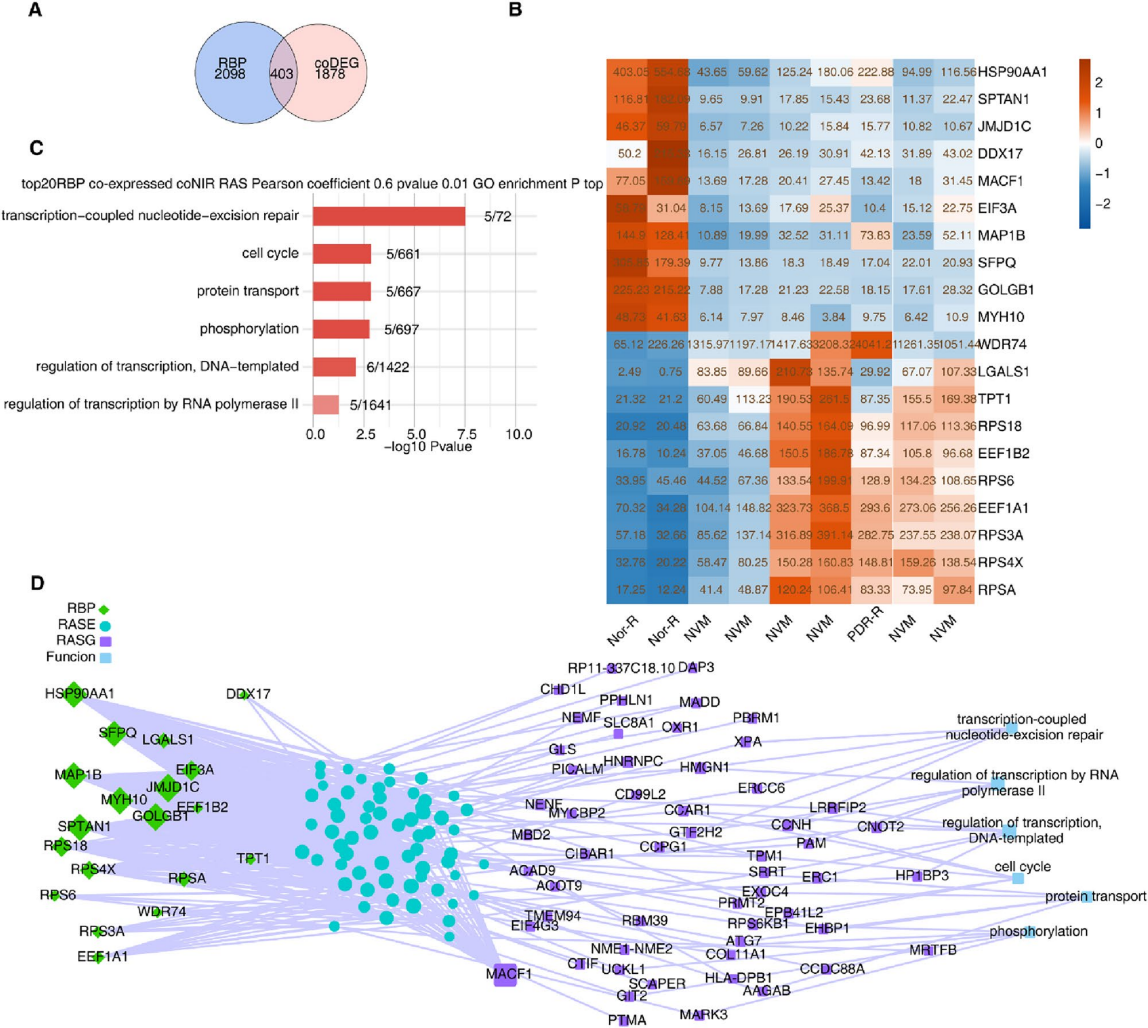
Co-expression analysis between RBPs and genes with alternative splicing in neovascular membranes and retinas from patients with proliferative diabetic retinopathy. (**A**) Venn diagram showing the overlap between co-expressed RBPs and DEGs. (**B**) Among the upregulated and downregulated coDE RBPs, RBPs with FDR ≤ 0.001 and the top 10 expression levels were screened, respectively, and the heatmap shows the expression levels of the top 20 RBPs. (**C**) Top 20 RBPs and coNIR RAS were co-expressed. Bar plot showing the most enriched GO biological processes of the top 20 RBP-regulated coNIR RAS. Cutoffs of *P*-value ≤ 0.01 and Pearson coefficient ≥ 0.6 or ≤  − 0.6 were applied to identify the co-expression pairs. (**D**) Network diagram showing the pathway of ASEs regulated by the top 10 RBPs. ASEs, alternative splicing events; coNIR, co-expressed non-intron retention; DEGs, differentially expressed genes; FDR, false discovery rate; GO, Gene Ontology; RAS, regulated alternative splicing; RBPs, RNA-binding proteins.

After overlapping the co-expressed RBP genes in NVM and PDR-R with the potential RBP genes in humans, we identified 403 DE RBPs (Fig. 3A). We further screened the top 20 DE RBPs, including heat-shock protein 90-alpha (HSP90AA1) and SFPQ, based on their FPKM values. These RBPs were abnormally expressed in NVM and PDR-R, suggesting that they regulated the AS of downstream genes (Fig. 3B). Considering the FPKM values of these RBPs in the three groups, we performed a co-expression analysis based on the PSI values of the co-expressed DE ASEs. We identified the co-expressed DE ASEs among these DE RBPs. GO functional analysis results showed that the co-expressed ASGs were enriched in transcription-coupled nucleotide-excision repair; cell cycle; protein transport; phosphorylation; regulation of transcription, DNA-templated; and regulation of transcription by RNA polymerase II (Fig. 3C). An interaction network between DE RBPs and ASEs was constructed, which implied that these DE RBPs may affect the AS of downstream genes (Fig. 3D). The most enriched KEGG pathways of the top 20 RBP-regulated co-expressed NIR (coNIR) AS are displayed in Supplementary Fig. 5.

### Abnormal RBPs potentially affect the AS of cell cycle-associated genes in NVM and retinas of patients with PDR

**Figure 4:**
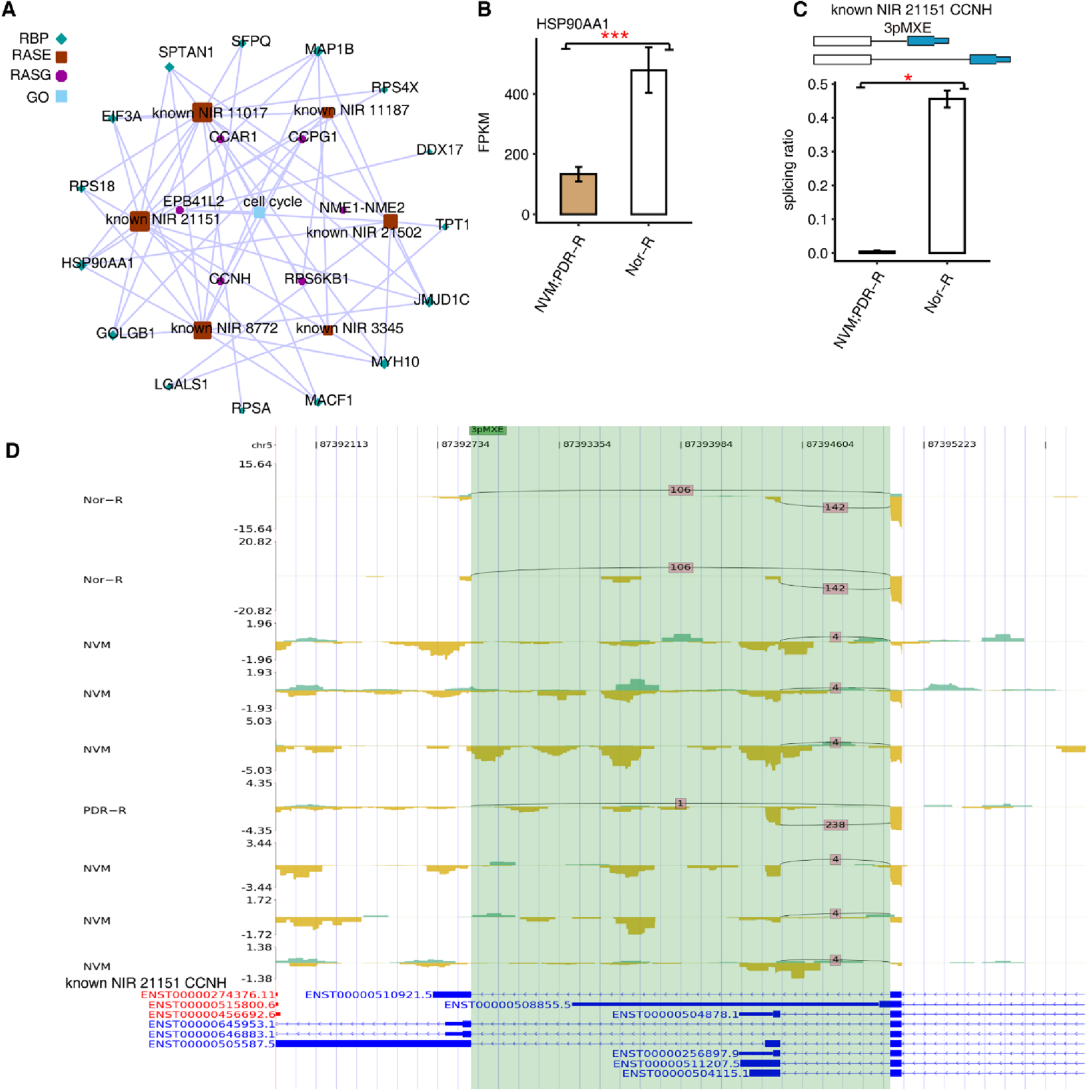
HSP90AA1 regulated CCNH in the cell cycle. (**A**) Network diagram of co-expression of differential alternative splicing events between RBPs and cell cycle. (**B**–**C**) Bar diagram showing expression of HSP90AA1 and mutually exclusive 3ʹ-UTRs (3PMXE) event of CCNH. #: not significant, *P < 0.05, **P < 0.01, ***P < 0.001. The exon sequences are denoted by boxes and intron sequences by the horizontal line. (**D**) Reads distribution chart showing CCNH. CCNH, CyclinH; HSP90AA1, heat-shock protein 90-alpha; RBPs, RNA-binding proteins.

We retrieved ASEs related to the cell cycle and their co-expressed RBPs and then constructed a co-expressed network. We found that DE RBPs, such as HSP90AA1 and SFPQ, may regulate downstream genes, such as CyclinH (CCNH) and cell cycle and apoptosis regulator 1 (CCAR1), which are highly relevant to the AS during the cell cycle (Fig. 4A). The bar graphs of HSP90AA1 and CCNH are shown in Fig. 4B,C, respectively, whereas the reads distribution chart of CCNH is displayed in Fig. 4D. A bar diagram showing that SFPQ regulates CCAR1 in the cell cycle is illustrated in Supplementary Fig. 6.

### Validation of DE RBPs and ASEs using an OIR mouse model

**Figure 5:**
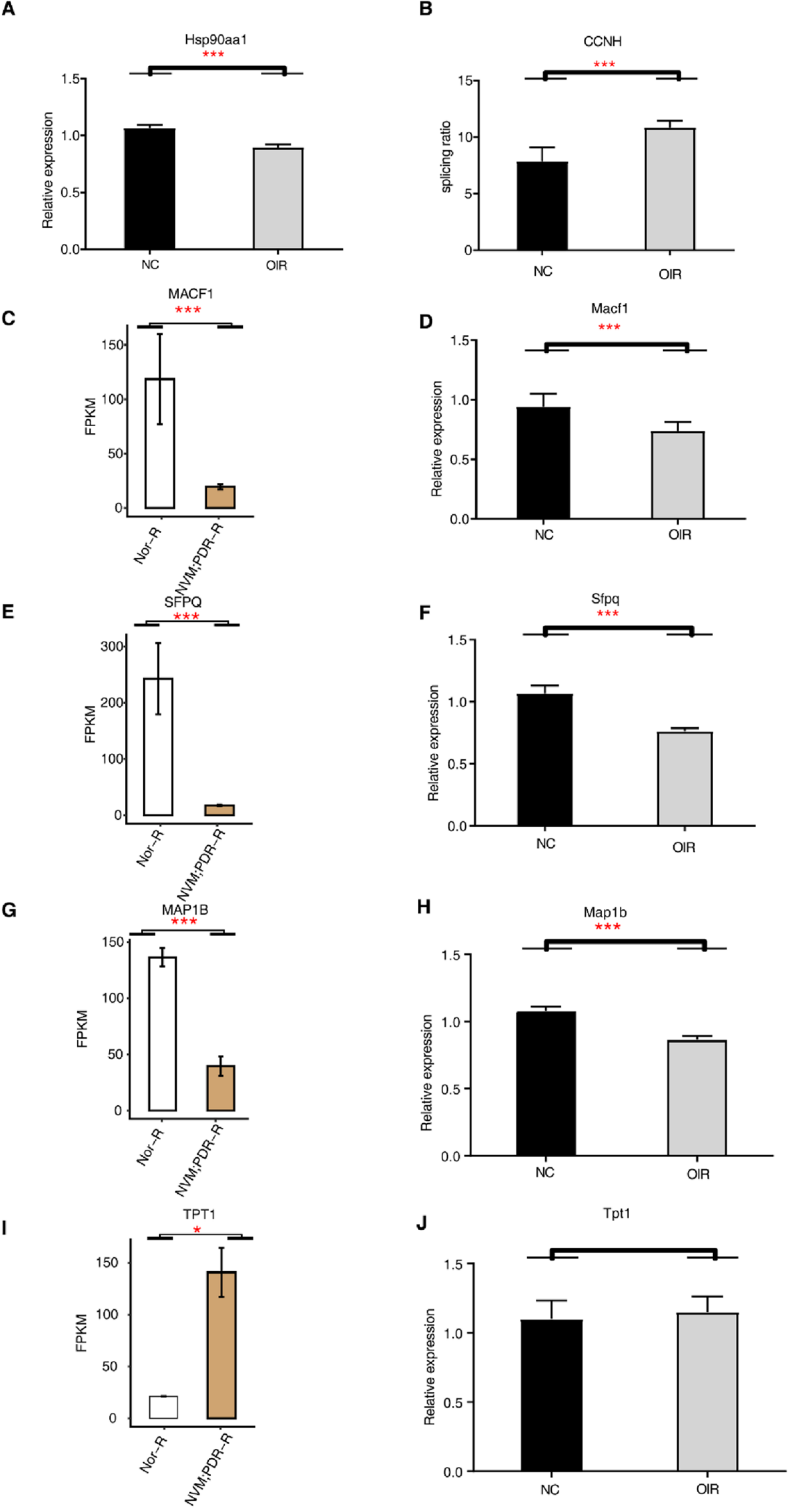
Validation of DE RBPs and ASEs in the OIR mouse model. (**A**,**D**,**F**,**H**,**J**) RT-PCR analysis for DE RBPs (Hsp90aa1, Macf1, Sfpq, Map1b, and Tpt1) in the retinas of OIR mice at P17. (**B**) RT-PCR analysis for DE ASEs (CCNH) in the retinas of OIR mice at P17. (**C**,**E**,**G**,**I**) Bar diagram showing MACF1, SFPQ, MAP1B, and TPT1. *P < 0.05, ***P < 0.001. ASEs, alternative splicing events; CCNH, CyclinH; DE RBPs, differentially expressed RNA-binding proteins; OIR, oxygen-induced retinopathy; MACF1, microtubule-actin crosslinking factor 1; MAP1B, microtubule-actin crosslinking factor 1; SFPQ, splicing factor proline/glutamine rich; Tpt1, tumor protein translationally controlled 1.

**Figure 6:**
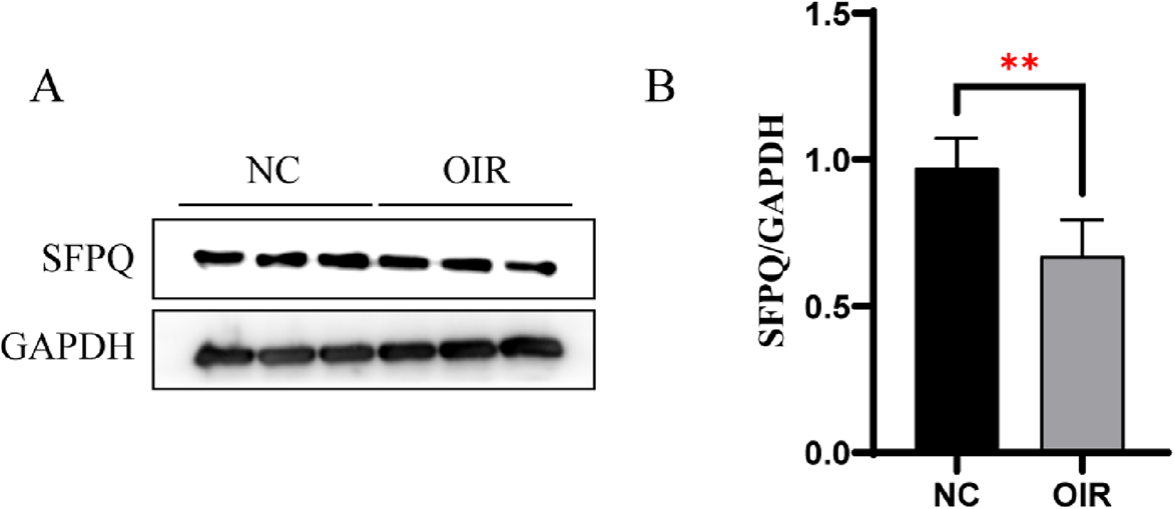
The protein expression of SFPQ by western blot (WB). (**A**) The representative image of WB. (**B**)The bar chart shows the protein expression level of SFPQ of the two groups. **P < 0.01. OIR, oxygen-induced retinopathy; SFPQ, splicing factor proline/glutamine rich.

An OIR mouse model was constructed to validate the role of the key genes in PDR. The expression of key genes was verified via reverse transcription polymerase chain reaction (RT-qPCR), and results showed that the expression levels of the key DE RBPs, namely, Hsp90aa1, microtubule-actin crosslinking factor 1 (MACF1), SFPQ, and microtubule-associated protein 1 B (MAP1B) and DE ASEs (CCNH) were significantly different between the retinas of the OIR and normal mice (*P* < 0.05; Fig. 5). However, the expression of tumor protein translationally controlled 1 (Tpt1) showed no significant change (*P* = 0.9883). All raw data with Ct values of the samples and controls are shown in Supplementary Table 2. Furthermore, WB was used to identify SFPQ protein expression. The outcomes show that SFPQ was down-regulated in retinas of OIR mice compared with retinas of WT mice (Fig. 6). The original, unprocessed electrophoretic gel images are included in Supplementary Fig. 7.

## Discussion

DR is the most common microvascular complication of diabetes and may lead to severe vision loss and heavy financial burden. Thus, treatment methods to delay or block the progression from non-proliferative DR to PDR are valuable. Li et al. showed that the transcriptional profiles of NVM are distinct from those of the retinas of patients with PDR^19^. The DEGs between PDR-R and NVM may be involved in the progression of NVM in PDR. Our study focused on the overlapping DEGs in PDR-R and NVM. GO functional analysis indicated that differentially downregulated genes were enriched in the RNA splicing pathway, mRNA processing, and mRNA splicing via spliceosome, suggesting that these genes regulate PDR progression by affecting RNA splicing.

RBPs are involved in complex interrelated molecular mechanisms of PDR, suggesting their potential as therapeutic targets for PDR^27^. Overexpression of the RBP lin-28 homolog B could exacerbate angiogenesis and elevate the protein levels of proangiogenic factors in human retinal endothelial cells and human retinal microvascular endothelial cells^28^. HuR may exacerbate DR by binding to and increasing VEGF expression^29^. Accumulating evidence supports that the dysregulation or dysfunction of RBPs can lead to PDR. In the present study, we screened the top 20 DE RBPs that may participate in the progression of DR. LGALS1, a beta-galactoside-binding protein, affects angiogenesis during DR, and inhibition of its expression could suppress DR progression^30,31^. Downregulation of SFPQ may promote tumor growth by increasing angiogenesis and interfering with the cell cycle^32^. MACF1 has been associated with neurodevelopmental and neurodegenerative diseases and plays a neuroprotective role in the optic nerve^33^. MAP1B plays an essential role in the development and functioning of the nervous system^34^. TPT1 contributes to retinal circuity formation, and its transcripts are enriched in embryonic retinal ganglion cells^35^. The roles in PDR progression of many of the RBPs mentioned above have not been studied systemically, and these RBPs may serve as targets in PDR treatments.

In the present study, few ASEs in normal retinas differed from those in PDR-R and NVM, which could indicate PDR progression. GO enrichment analysis showed that the potential signaling pathways of ASEs included transcription-coupled nucleotide-excision repair, mRNA processing, and cell cycle. The cell cycle plays an important role in PDR development^36–38^. Kim et al. found that the population of human umbilical vein endothelial cells gradually increases in the G2/M phase and decreases in the G0/G1 and S phases after exposure to homoisoflavanone, which inhibits RNV^36^. Rosmarinic acid prevents the proliferation of retinal endothelial cells in a dose-dependent manner by causing cell cycle arrest in the G2/M phase^37^. Arctiin decreases the proliferation of high glucose-induced human retinal capillary endothelial cells by inducing cell cycle arrest in the G0/G1 phase^38^.

The differential expression of RBPs and ASEs between the NVM, Nor-R, and PDR-R groups was analyzed to establish an RBP–AS co-expression network. Results revealed the potential mechanism of RBPs in PDR pathogenesis. The top 20 RBP co-expressed AS genes were enriched in cell cycle pathways. We further extracted cell cycle-related RASEs. A co-expression analysis was conducted to explore the regulatory and interaction relationship between selected RBPs (SFPQ, MAP1B, RPS4X, DDX17, TPT1, JMJD1C, MYH10, MACF1, RPSA, LGALS1, GOLGB1, HSP90AA1, RPS18, EIF3A, and SPTAN1) and their regulated ASGs (CCAR1, CCPG1, RPS6KB1, CCNH, and EPB41L2). These RBPs modulate their corresponding ASGs possibly by regulating the cell cycle during DR progression.

CCPG1 may participate in cell proliferation and apoptosis in the retinoblastoma^39^. RPS6KB1 affects the development of colorectal cancer by regulating the cell cycle^40^. EPB41L2 is a membrane scaffold protein that mediates membrane transport, which is critical for the correct placement of rod synapses in the retina and normal visual function^41^. CCNH regulates the cell cycle by modulating the activity of cyclin-dependent kinase 7^42^. CCNH may also affect the cell cycle by participating in mitosis^43^. The present study showed that the splicing ratio of 3pMXE event of CCNH in Nor-R was significantly higher than that in NVM and PDR-R. Whether there is a correlation between the different splicing ratio of CCNH and its regulatory function on the cell cycle is worth further investigation. CCAR1 is a cyclin, and inhibition of its expression can suppress the proliferation and migration of cancer cells^44^. Alternative RNA splicing contributes to divergent CCAR1 activities^45^.

Proteomic analysis of the human retina showed that HSP90AA1 is highly expressed in normal retinal tissue, which is consistent with our sequencing results^46^. In the present study, HSP90AA1 expression was downregulated in the retinas and NVM of patients with PDR, indicating that HSP90AA1 participates in PDR by regulating the AS of CCHN. As a splicing factor in the network that we constructed, SFPQ can be recruited by the lncRNA NEAT1 and then bind directly with the mRNA of MITF to regulate its AS. SFPQ overexpression in ARPE-19 cells enhances the binding affinity of SFPQ to MITF and the splicing efficiency of ( +) MITF^17^. The results of the present study indicated that SFPQ can regulate PDR progression by influencing the AS of CCAR1. To confirm these results, we constructed an OIR mouse model. OIR models are used to study specific pathological events that commonly occur during DR^47^. SFPQ, MAP1B, HSP90AA1, MACF1, and CCNH were significantly DE in the OIR mice. The results suggest that these genes participate in PDR progression. Investigating the RBP–AS interaction network using different models of PDR may resonate with our findings. We selected target gene SFPQ for further validation by western blot in the OIR model. Both RNA and protein expression of SFPQ showed the same down-regulated tendency in OIR. What effect does overexpression of SFPQ have on PDR progression remain to be explored. Furthermore, the potential functions of the verified RBPs and the connections between RBPs and ASGs warrant further investigations.

Our study has some limitations. First, the NVM and retina are different tissues with specific cell types that are present in different proportions. The genes differentially expressed between these samples will largely reflect the different cell types present. Single-cell RNA-sequencing is necessary to further explore the gene expression and mechanisms involved in PDR pathogenesis. Second, the use of mouse OIR models cannot completely simulate the pathophysiological changes in PDR. Particularly, the limitation that the OIR model does not show neovascular membrane may lead to the discrepancy between laboratory findings and clinical findings of PDR. Therefore, future RNAseq of the OIR model should be investigated, as it may reveal the connections between the splicing events and shed light on how well the model fits the human PDR dataset. Additionally, our findings could be expanded by future research using several different DR models, such as db/db mouse model. Third, the GSE data selected for analysis were human samples, but mouse retinas were used for verification. This discrepancy might explain why the Ct values of the primers for a few DE ASGs (CCAR1 and CCPG1) were too high to be measured. Certain genes are characterized by striking differences between species, although global patterns of transcript diversity are similar between the human and mouse cortices^48^. Species-specific transcriptional complexity cannot be ignored in fundamental and clinical conversion.

In conclusion, this study analyzed retina and NVM samples from patients with PDR by using data from the GEO database and identified many DE RBPs and ASEs. A co-expression network between abnormal RBPs and DE ASEs was established. RBPs, such as HSP90AA1 and SFPQ, may affect PDR development by modulating the ASEs of cell cycle-associated downstream genes, such as CCNH or CCAR1. These findings provide evidence of the importance of RBPs and ASEs in PDR pathogenesis. This study may serve as a basis for discovering novel targets and developing novel treatment strategies against retinal angiogenic diseases, such as PDR.

### Supplementary Information

Supplementary Information.

## Data Availability

The datasets generated/analyzed for this study can be found in the public transcriptome dataset GSE102485 from the Gene Expression Omnibus (GEO) database (https://www.ncbi.nlm.nih.gov/geo/). Other data that support the findings of this study are available in the supplementary material of this article.
