# Supplementary material for: RNA-binding proteins potentially regulate the alternative splicing of cell cycle-associated genes in proliferative diabetic retinopathy
Source: Sci Rep. 2024 Mar 20;14:6731. doi: 10.1038/s41598-024-57516-x (PMC10954754; doi:10.1038/s41598-024-57516-x)
Supplement: Supplementary file 1 — Supplementary Information. [file 41598_2024_57516_MOESM1_ESM.pdf]

Supplementary Material

1 Supplementary Figures and Tables

1.1 Supplementary Figures

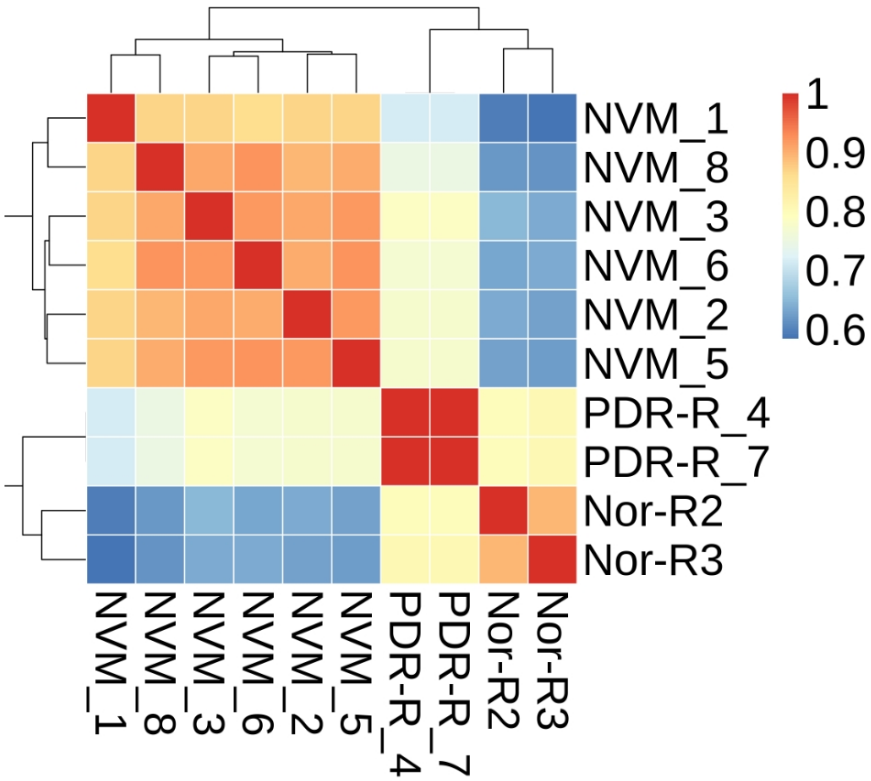

Supplementary Figure 1. Sample correlation and cluster analysis. NVM, neovascular membranes; PDR-R, retinas from patients with proliferative diabetic retinopathy; Nor-R, normal retinas from donated eyes of normal individuals.

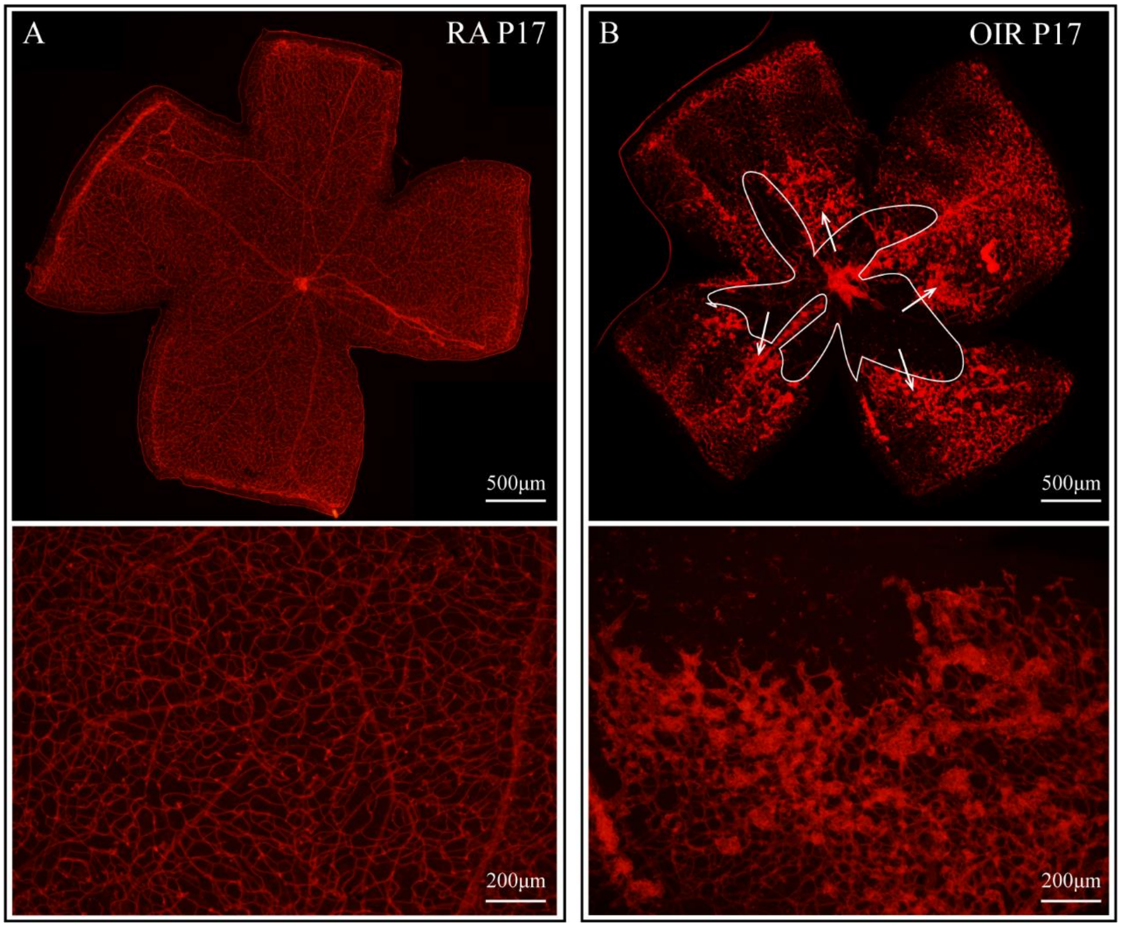

Supplementary Figure 2. Retinal hypoxic condition in OIR at P17. Flat-mount retinal vasculatures stained by isolectin B4 (red) show the construction of retinal vessels. (**A**) The RA group. (**B**) The OIR group. Large nonperfused zones (white irregular circle) and retinal neovascularisation (white arrows) were discovered in the OIR group at P17. (magnification 40×; scale bar = 500 μm in the upper panels; magnification 100×; scale bar = 200 μm in the lower panels). OIR, oxygen-induced retinopathy; RA, room air.

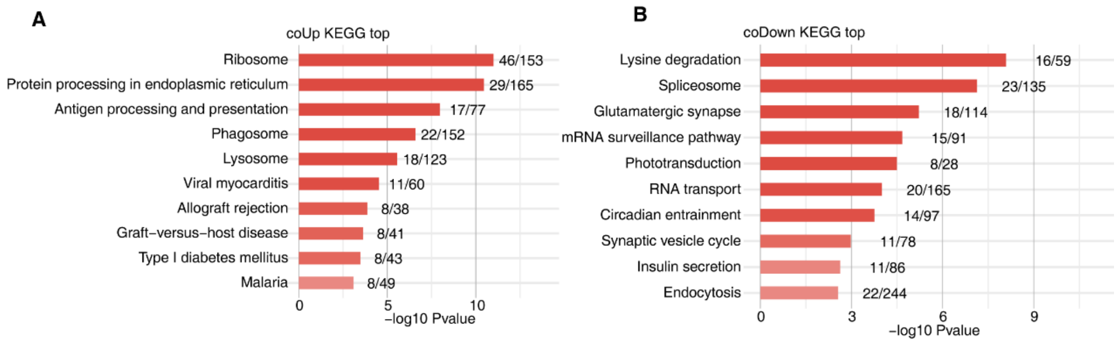

Supplementary Figure 3. Gene expression profile of neovascular membranes and retinas from patients with proliferative diabetic retinopathy. (**A**) Bar plot showing the most enriched KEGG

pathways for all co-upregulated DEGs. (**B**) Bar plot showing the most enriched KEGG pathways for all co-downregulated DEGs. DEGs, differentially expressed genes; KEGG, Kyoto Encyclopedia of Genes and Genomes.

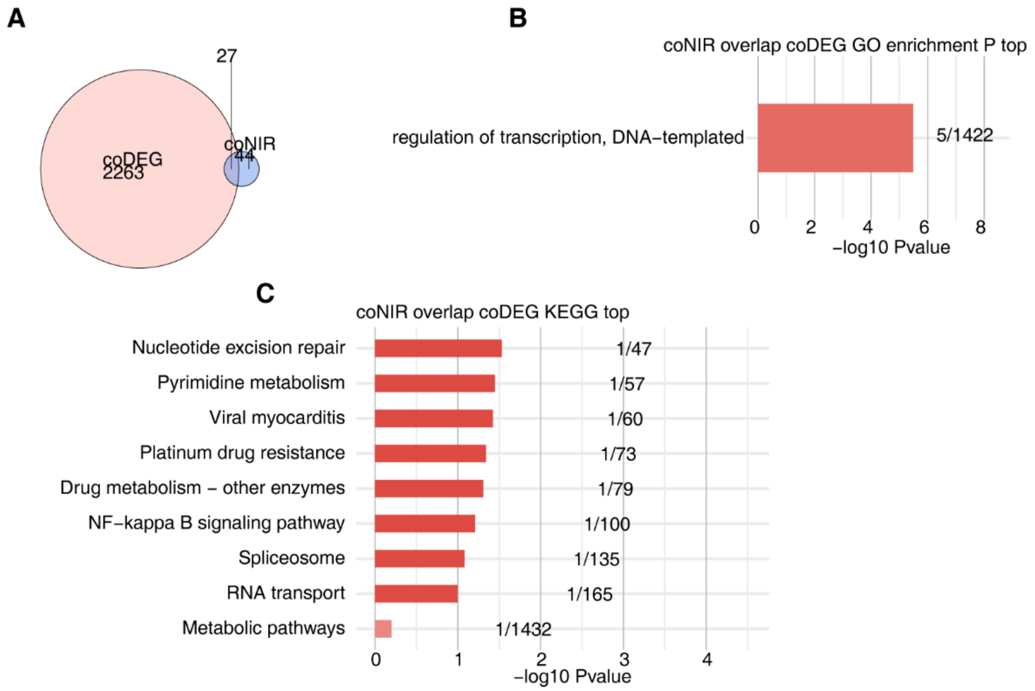

Supplementary Figure 4. Abnormal alternative splicing pattern of genes in neovascular membranes and retinas from patients with proliferative diabetic retinopathy. (**A**) Using the gene symbol, a Venn diagram showing the overlap of coDEGs and coNIR between samples from the NVM, PDR-R, and Normal groups. (**B**) Bar plot showing the most enriched GO biological processes of overlapped coDEGs and coNIR between samples from the NVM and PDR-R groups. (**C**) Bar plot showing the most enriched KEGG pathway results of the overlapped coDEGs and coNIR between samples from the NVM and PDR-R groups. coDEGs, co-expressed differentially expressed genes; coNIR, co-expressed non-intron retention; GO, Gene Ontology; KEGG, Kyoto Encyclopedia of Genes and Genomes; NVM, neovascular membranes; PDR-R, retinas from patients with PDR.

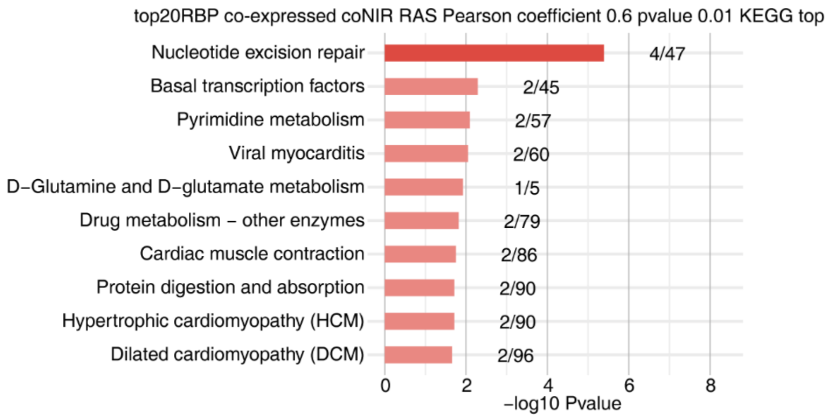

Supplementary Figure 5. Co-expression analysis between RBPs and genes with alternative splicing in neovascular membranes and retinas from proliferative diabetic retinopathy. Bar plot showing the most enriched KEGG pathway results of the top 20 RBP-regulated coNIR RAS. Cutoffs of p-value ≤ 0.01 and Pearson coefficient ≥ 0.6 or ≤ -0.6 were applied to identify the co-expression pairs. coNIR, co-expressed non-intron retention; KEGG, Kyoto Encyclopedia of Genes and Genomes; RAS, regulated alternative splicing; RBPs, RNA-binding proteins.

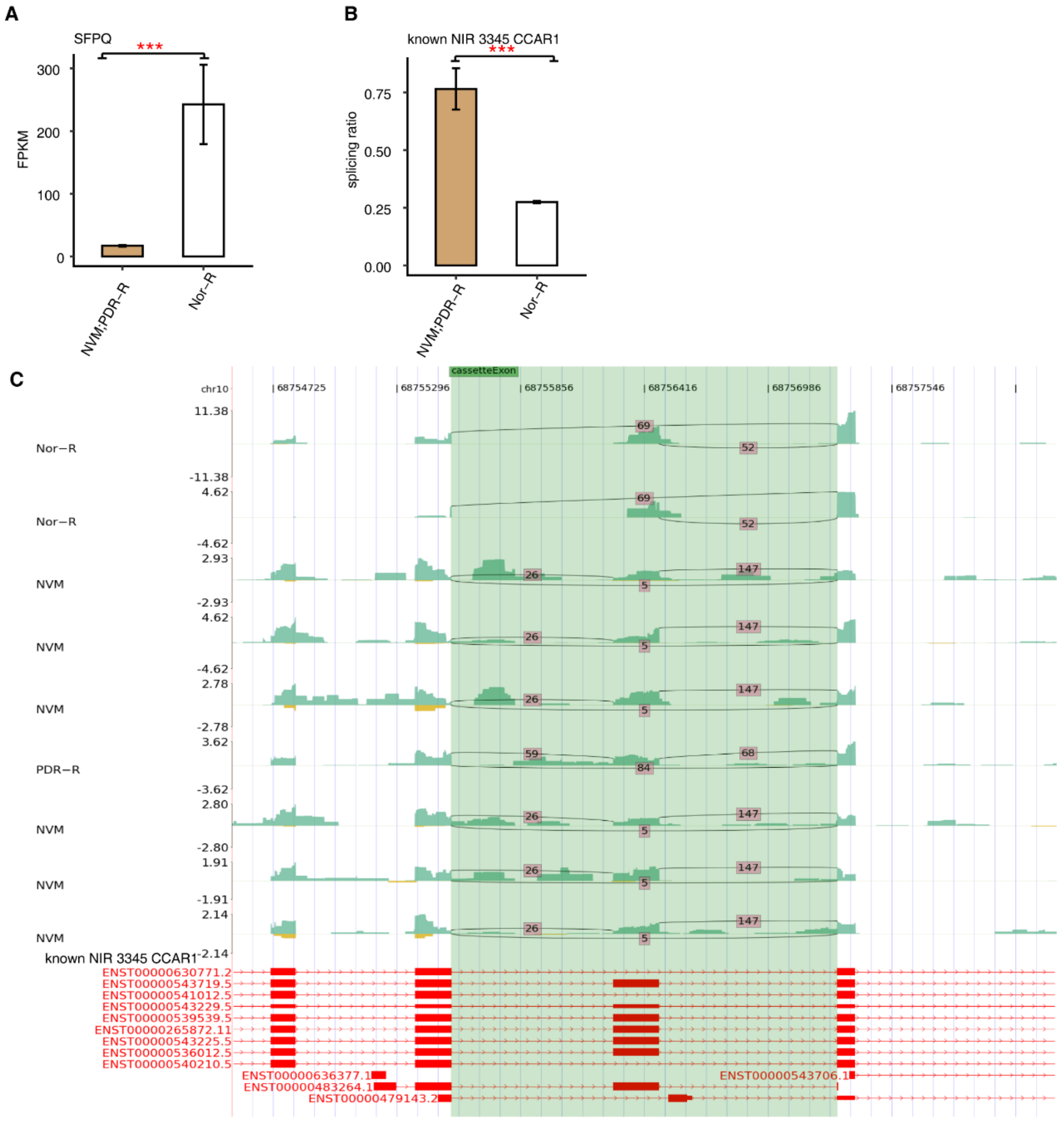

Supplementary Figure 6. SFPQ regulated CCAR1 in the cell cycle. (**A**–**B**) Bar diagram showing HSP90AA1 and CCNH expression. #: not significant, *p < 0.05, **p < 0.01, ***p < 0.001. (**C**) Reads distribution chart showing CCNH.

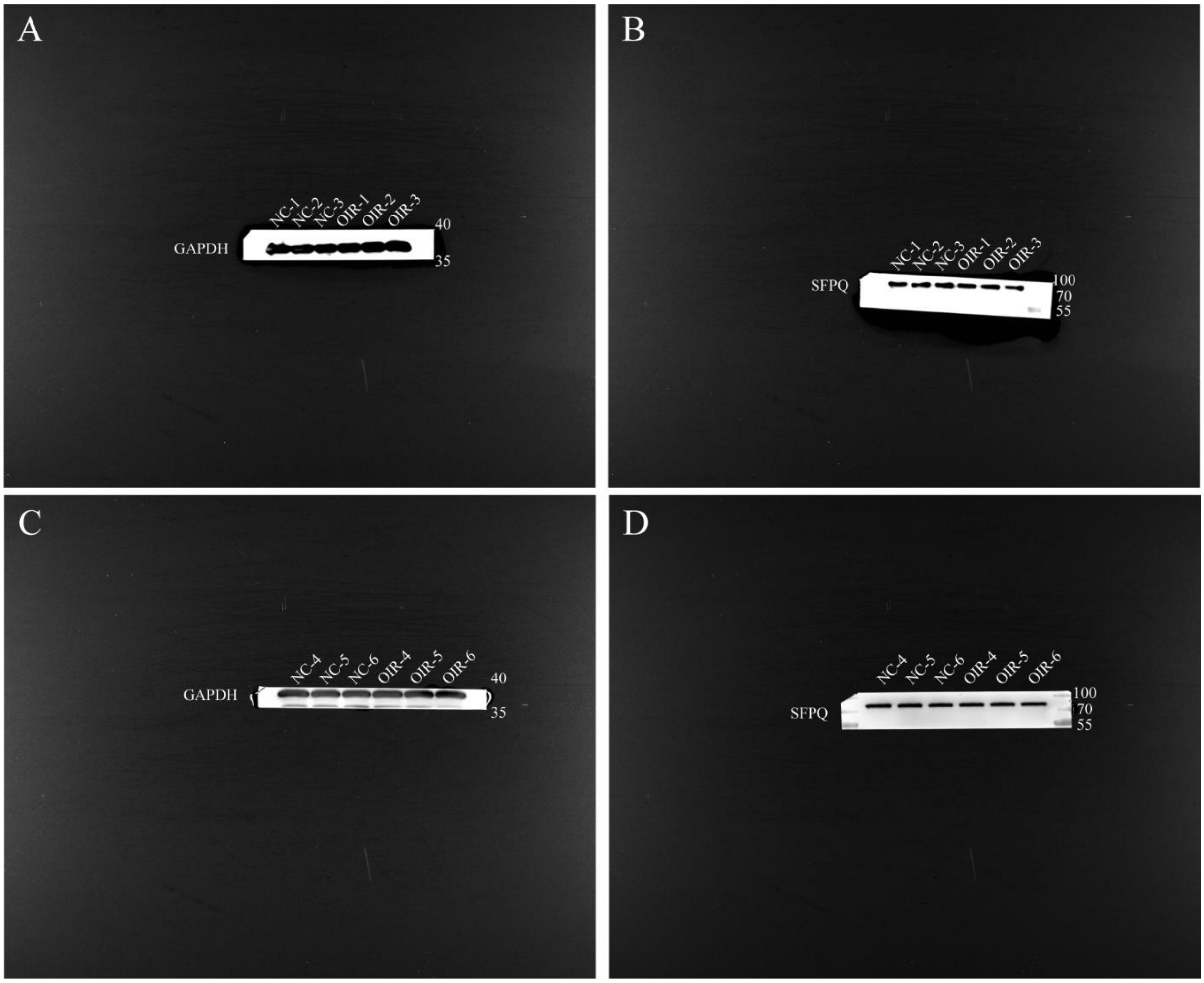

Supplementary Figure 7. The protein expression of SFPQ by western blot. (**A**–**D**) The original, unprocessed electrophoretic gel images.

1.2 Supplementary Tables

Supplementary Table 1. Sample descriptions

ID	Name	Description	Average Age (Y)	Average course of disease (Y)
GSM2739342-45	NVM_1	disease: T2D (V); tissue: hemal arch part	46.5	9
GSM2739346-48	NVM_2	disease: T2D (V); tissue: nasal side	43	4.33
GSM2739349-52	NVM_3	disease: T2D (V); tissue: whole part	47.75	7.25

GSM2739355-56	NVM_5	disease: T2D (V); tissue: hemal arch part and optic disc	46	11
GSM2739357-58	NVM_6	disease: T2D (V); tissue: optic disc part	47	11
GSM2739359-60	PDR-R_7	disease: T2D (V); tissue: surrounding part	48	10.5
GSM2739361-63	NVM_8	disease: T1D (V); tissue: whole part	27.66	14
GSM2739365	Nor-R2	disease: Normal retina; tissue: retina	--	--
GSM2739366	Nor-R3	disease: Normal retina; tissue: retina	--	--

Type I diabetes, T1D; Type II diabetes, T2D; “V” represents the fifth stage of diabetic retinopathy; “--” represents not applicable.

Supplementary Table 2. Cts of samples and controls

Sample Name	Ct	Ct reference	Ct mean
NC-1	23.404	14.109	14.08333
	23.645	14.037	
	23.464	14.104	
NC-2	24.265	13.911	13.91233
	23.903	13.908	
	24.071	13.918	
NC-3	23.834	14.327	14.177
	23.741	14.079	
	23.725	14.125	
NC-4	23.497	14.085	14.049
	23.398	14.020	
	23.524	14.042	
OIR-1	23.200	13.907	13.94133
	22.980	13.931	
	23.265	13.986	
OIR-2	22.984	13.727	13.64767
	23.040	13.647	
	23.258	13.569	
OIR-3	22.810	13.754	13.755
	22.739	13.805	
	22.629	13.706	
OIR-4	23.072	13.758	13.63867
	22.953	13.381	

	22.988	13.777	
